# Prognostic value of ABO blood groups in upfront operated pancreatic ductal adenocarcinomas

**DOI:** 10.1007/s00423-024-03469-8

**Published:** 2024-09-18

**Authors:** Gaëtan-Romain Joliat, Ismail Labgaa, David Martin, Dionisios Vrochides, Markus Schäfer

**Affiliations:** 1 Department of Visceral Surgery, Lausanne University Hospital CHUV, University of Lausanne (UNIL), Rue du Bugnon 46, Lausanne, 1011 Switzerland; 2 Graduate School of Health Sciences, University of Bern, Bern, Switzerland; 3 Department of Surgery, Division of Hepatobiliary and Pancreatic Surgery, Carolinas Medical Center, Charlotte, USA

**Keywords:** Prognosis, Pancreas cancer, Survival, Pancreatoduodenectomy, Pancreatectomy

## Abstract

**Purpose:**

Pancreatic ductal adenocarcinoma (PDAC) has been shown to have a lower incidence in patients with blood group O. It is currently uncertain if patients with group O have a better prognosis after pancreatectomy. This study assessed the overall survival (OS) and disease-free survival (DFS) of PDAC patients who underwent upfront pancreatoduodenectomy based on ABO blood groups.

**Methods:**

A cross-sectional study was performed including patients from two university centers. All consecutive head PDAC patients who underwent upfront pancreatoduodenectomy from 2000 to 2016 were included. OS and DFS were compared between blood groups A, B, AB, and O using Kaplan-Meier curves and log-rank tests.

**Results:**

A total of 438 patients were included (215 women, median age 67). Pre- and intraoperative details were comparable between all subgroups. Median OS did not differ between the four blood groups (A: 23 months, 95% CI 18–28; B: 32, 95% CI 20–44; AB: 37, 95% CI 18–56 and O: 26, 95% CI 20–32, *p* = 0.192). Median DFS were also similar (A: 19 months, 95% CI 15–23; B: 26, 95% CI 19–33; AB: 35, 95% CI 15–55 and O: 22, 95% CI 15–29, *p* = 0.441). There was no OS difference between O and non-O groups (median: 26 months, 95% CI 20–33 vs. 25 months, 95% CI 20–30, *p* = 0.773). On multivariable analysis blood groups were not prognostic of OS. Only lymph node involvement, tumor differentiation, and adjuvant chemotherapy were independent prognostic factors.

**Conclusion:**

OS and DFS were similar between all four blood groups after pancreatoduodenectomy. Independent predictors of OS were associated with tumor characteristics and adjuvant treatment.

## Introduction

Pancreatic ductal adenocarcinoma (PDAC) is the most frequent type of pancreatic cancers [1]. Even when operated, PDAC patients have a poor prognosis with median overall survival (OS) of around 20 months [2]. Several predictive factors of OS of PDAC patients have been described such as lymph node involvement, tumor differentiation, resection status margin, or early recurrence [3].

In pancreatic cancer, types of ABO blood groups have recently been shown to have an influence on the incidence rate. Based on data from two large US cohorts, patients (*n* = 107’503) with A, B, or AB groups have been shown to be associated with a higher incidence of pancreas cancer compared to patients with O group (HR 1.32, HR 1.72, and HR 1.51, respectively) [4]. Several additional studies have confirmed these results in different populations showing that patients with O group had less risk of developing pancreas cancer [5–8]. A recent US study showed that blood groups did not influence the patient outcomes after pancreatectomy for pancreas cancer [9], but data on survival after resection and blood groups remain scarce for PDAC.

The aim of the present study was to assess the OS and disease-free survival (DFS) based on the ABO blood group types in a cohort of patients with resectable ductal adenocarcinoma of the pancreatic head who were operated upfront.

## Materials and methods

### Patients and centers

A cross-sectional study was performed. All consecutive patients with PDAC of the pancreas head who were operated between January 2000 and December 2016 were included. As PDAC of the tail of the pancreas have different prognosis and survival than PDAC of the pancreatic head [10, 11], only tumors of the pancreatic head were included to have a homogeneous cohort. Two centers participated to this study: Lausanne University Hospital CHUV (Lausanne, Switzerland) and Carolinas Medical Center (Charlotte, United States of America). This study was approved by the Institutional Review Board (CER-VD #2017 − 01169). All data were retrospectively collected.

### Eligibility criteria

Only patients who underwent upfront pancreatoduodenectomy for resectable PDAC were included. Patients with borderline or locally advanced tumors or with PDAC of the pancreatic tail were excluded. Exclusion criteria were other histology types, neoadjuvant treatment, or refusal of the patients to use their data for research. Patients with metastases at diagnosis, with macroscopic positive resection (R2), or who died during the first 90 postoperative days were also excluded.

### Surgery and definitions

Pylorus-preserving or classic pancreatoduodenectomy were performed according to the surgeon’s preference.

Bloods groups were defined before the operation using a regular blood test. Blood groups were classified into the following categories: A, B, AB, and O. Rhesus factors were also considered (positive and negative). Postoperative complications until 90 days after surgery were defined based on the Clavien classification [12]. The Comprehensive Complication Index (CCI, from 0 = no complication to 100 = death) was used to summarize the total burden of complications per patient [13]. Recurrence was based on postoperative imaging or histology. Recurrence encompassed local disease progression and distant metastases. Length of stay was defined from the day of the operation until hospital discharge. Decision for adjuvant chemotherapy was based on a multidisciplinary tumor board decision. R1 was defined as the presence of microscopic cancer cells within 1 mm of the surgical resection margin as defined by the Royal College of Pathologists (1-mm rule) [14].

### Statistics

Continuous data were summarized using median and interquartile range, and categorical data using frequency and percentage. Comparisons of continuous and categorical data were performed with Mann-Whitney U and chi-square tests, respectively. For comparisons of continuous data of more than two groups, a Kruskal-Wallis test was used. Median follow-up was calculated using the inverse Kaplan-Meier method. Survival curves were created with the Kaplan-Meier method and compared using the log-rank test. For the survival analysis, patients were censored once dead or lost to follow-up. Summary of survival analyses were presented as median in months with 95% confidence interval (CI). Predictive factors of survival were calculated using a multivariable Cox proportional hazard regression analysis. Items that had a p-value < 0.1 on univariable analysis were included in the multivariable analysis. A p-value < 0.05 was considered significant. Statistical analyses were performed using SPSS Statistics 29.0 for Mac OS X (Armonk, USA).

## Results

A total of 438 patients were included in the study (215 women, 223 men). A hundred and forty-two patients were from Lausanne University Hospital CHUV and 296 from Carolinas Medical Center. There was no difference in terms of preoperative characteristics and intraoperative data between both centers. Median age of the overall patient cohort was 67 (IQR 59–75). Postoperative complications occurred in 61% of patients (266/438) and median CCI was 8.7 (0-26.2). Median length of stay was 12 days (IQR 8–19). Within a median follow-up of 46 months (95% CI 41–51), median OS and DFS were 25 months (95% CI 20–30) and 22 months (95% CI 18–26), respectively.

### Blood groups

**Fig. 1:**
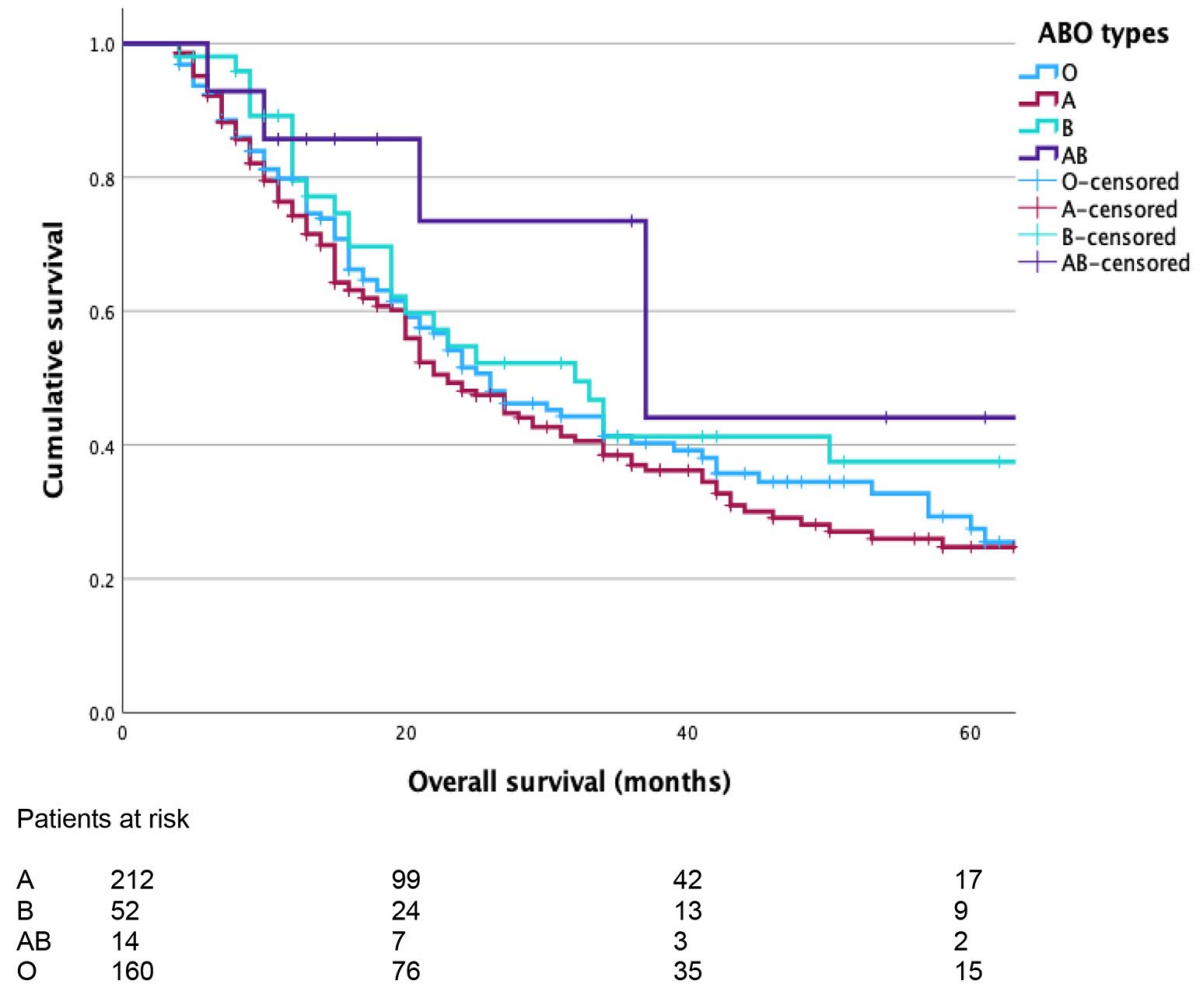
Kaplan-Meier curves comparing overall survival (OS) of patients based on their blood groups (median OS: 23 months, 95% CI 18–28 for the group A, 32 months, 95% CI 20–44 for the group B, 37 months, 95% CI 18–56 for the group AB, and 26 months, 95% CI 20–32 for the group O, log-rank test *p* = 0.192)

**Fig. 2:**
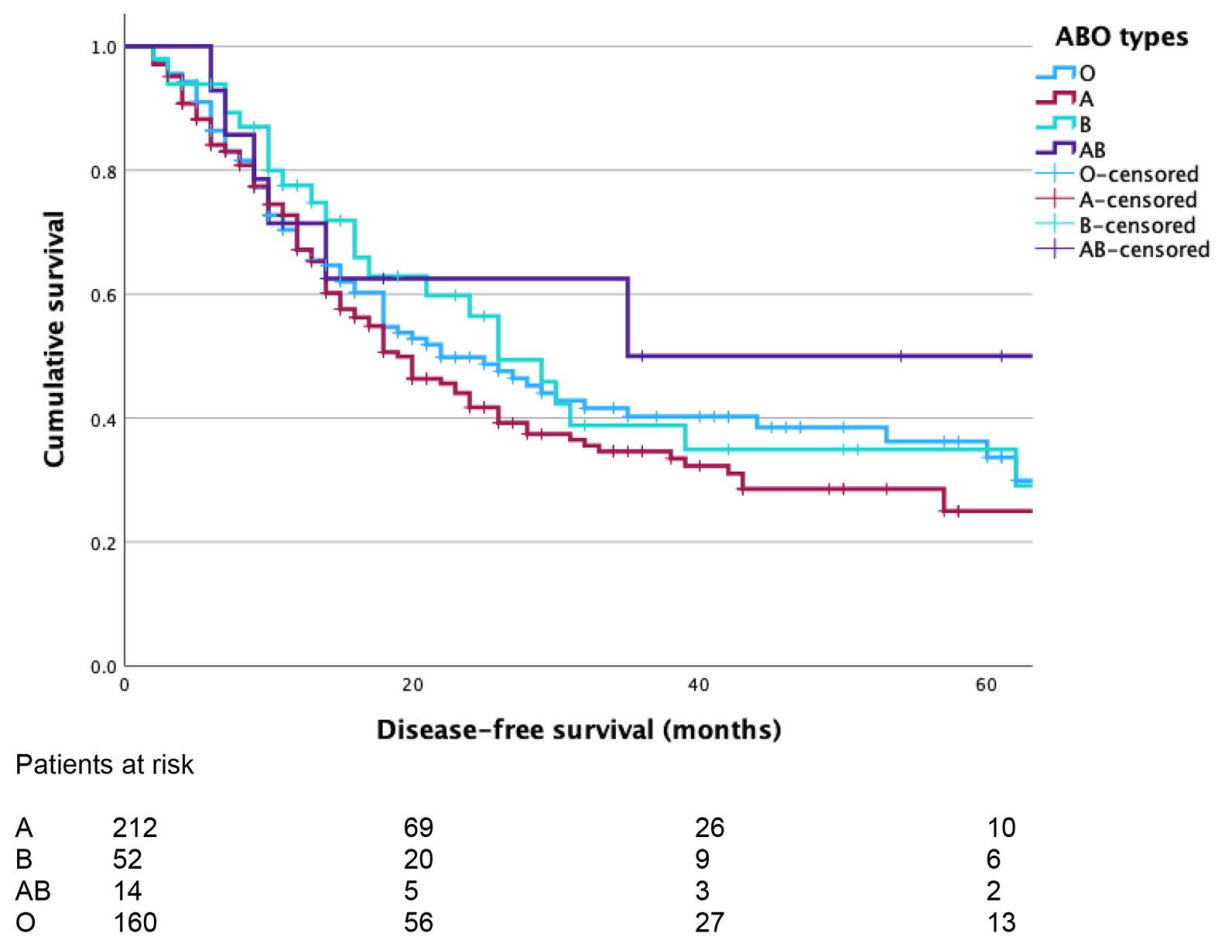
Kaplan-Meier curves comparing disease-free survival (DFS) of patients based on their blood groups (median DFS: 19 months, 95% CI 15–23 for the group A, 26 months, 95% CI 19–33 for the group B, 35 months, 95% CI 15–55 for the group AB, and 22 months, 95% CI 15–29 for the group O, log-rank test *p* = 0.441)

Blood groups were distributed as follows: A 212 patients (A + 170 patients), B 52 patients (B + 45 patients), AB 14 patients (AB + 12 patients) and O 160 patients (O + 135 patients). In total, 362 patients were Rhesus positive and 76 Rhesus negative. Characteristics, intraoperative, and pathological data of the patients based on the blood groups are summarized in Table 1. The rates of postoperative complications were similar in all subgroups of patients based on the ABO blood groups (A 97/212, B 32/52, AB 7/14, O 71/160, *p* = 0.172). OS was 23 months (95% CI 18–28) for the group A, 32 months (95% CI 20–44) for the group B, 37 months (95% CI 18–56) for the group AB, and 26 months (95% CI 20–32) for patients in group O (*p* = 0.192, Fig. 1). There was no OS difference between O and non-O groups (median: 26 months, 95% CI 20–33 vs. 25 months, 95% CI 20–30, *p* = 0.773). DFS was also similar between the four subgroups (A 19 months, 95% CI 15–23; B 26 months, 95% CI 19–33; AB 35 months, 95% CI 15–55, and O 22 months, 95% CI 15–29, *p* = 0.441, Fig. 2).

**Table 1:** Preoperative characteristics, intraoperative details and pathological results of the patients based on the different ABO blood groups

	Group A *N* = 212	Group B *N* = 52	Group AB *N* = 14	Group O *N* = 160	*P*-value
Age, *years*	67 (59–74)	65 (61–78)	69 (58–75)	68 (59–77)	0.705
Women	104 (49%)	26 (50%)	7 (50%)	78 (49%)	0.512
BMI, kg/m^2^	25 (23–29)	24 (22–28)	24 (21–29)	25 (23–29)	0.408
Smokers	47 (22%)	12 (23%)	2 (14%)	37 (23%)	0.897
Pre-existing DM	39 (18%)	13 (25%)	2 (14%)	29 (18%)	0.671
Preoperative biliary stent	139 (66%)	36 (69%)	11 (79%)	105 (66%)	0.745
ASA scores I-II	77 (36%)	17 (33%)	4 (29%)	44 (28%)	0.343
pT1-T2 stage	32 (15%)	12 (23%)	1 (7%)	32 (20%)	0.297
pN + stage	171 (81%)	37 (72%)	11 (79%)	110 (69%)	0.058
Grading G1-G2	133 (63%)	33 (63%)	10 (71%)	98 (61%)	0.895
R0 resection	137 (65%)	32 (62%)	11 (79%)	115 (72%)	0.283
Venous resection	43 (20%)	4 (8%)	2 (14%)	24 (15%)	0.145
Operation time, *min*	336 (277–413)	290 (245–363)	340 (246–412)	333 (267–397)	0.095
Intraoperative transfusion	57 (27%)	9 (17%)	4 (29%)	58 (36%)	0.470

Results are shown in median with interquartile range or number with percentage

BMI: body-mass index, DM: diabetes mellitus, ASA: American Society of Anesthesiologists

**Fig. 3:**
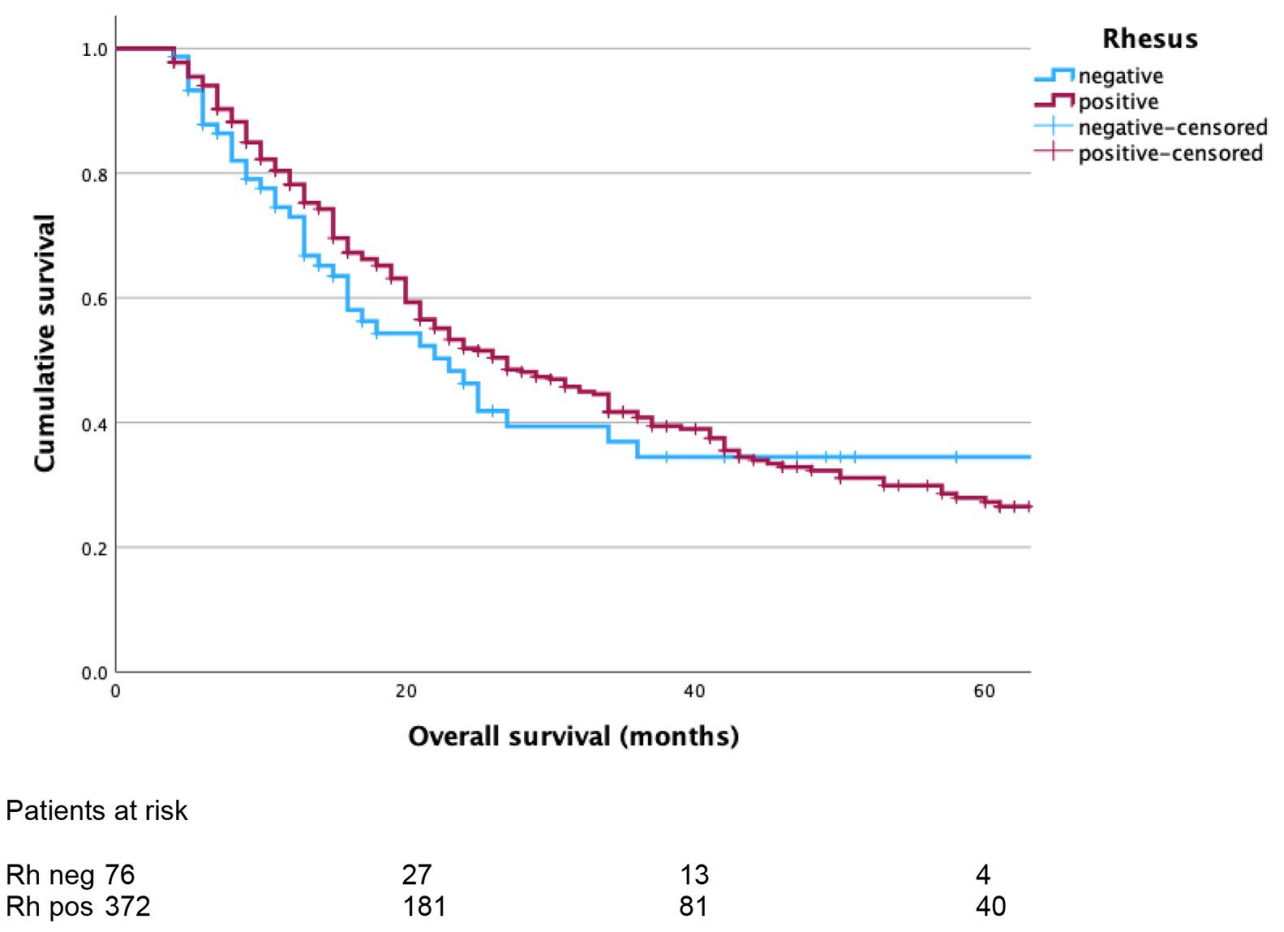
Kaplan-Meier curves comparing overall survival (OS) of patients with Rhesus negative and positive (median OS: 23 months, 95% CI 216 − 30 vs. 27 months, 95% CI 21–33, log-rank test *p* = 0.445)

Patients with Rhesus negative blood groups (*n* = 76) had similar median OS as patients with Rhesus positive blood groups (*n* = 372, 23 months, 95% CI 216 − 30 vs. 27 months, 95% CI 21–33, *p* = 0.445, Fig. 3). No difference of median DFS was found between both groups (16 months, 95% CI 8–24 vs. 23 months, 95% CI 19–27, *p* = 0.459).

### Predictors of survival

On multivariable Cox regression analysis, lymph node involvement, tumor differentiation, and adjuvant chemotherapy were found as predictor of OS. On the contrary, blood group was not found as a survival predictor. Table 2 shows the detailed results of the multivariable analysis.

**Table 2:** Multivariable Cox regression analysis of factors predictive of overall survival

	Univariable HR (95% CI)	*P*-value	Multivariable HR (95% CI)	*P*-value
Age	1.0 (1.0–1.0)	0.480		
Women	0.8 (0.6–1.1)	0.131		
BMI, kg/m^2^	1.0 (1.0–1.0)	0.247		
Active smokers	1.1 (0.8–1.5)	0.490		
Diabetes mellitus	1.2 (0.9–1.6)	0.318		
Preoperative biliary stenting	1.0 (0.8–1.3)	0.983		
ASA scores III/IV	1.0 (0.8–1.4)	0.877		
Blood groups Reference O A B AB	1 1.0 (0.8–1.3) 1.0 (0.7–1.5) 0.6 (0.3–1.4)	0.910 0.968 0.261		
pT stage Reference pT1 pT2 pT3 pT4	1 1.1 (0.5-2.0) 1.6 (0.9–2.8) 1.3 (0.6–2.9)	0.896 0.135 0.473		
pN stage Reference pN0 pN1 pN2	1 2.3 (1.6–3.3) 3.6 (2.5–5.3)	**< 0.001** **< 0.001**	2.6 (1.8–3.9) 4.1 (2.7–6.2)	**< 0.001** **< 0.001**
Tumor grading Reference G1 G2 G3	1 1.0 (0.7–1.5) 1.7 (1.1–2.6)	0.958 **0.012**	1.7 (1.1–2.6)	**0.025**
R1 resection	1.4 (1.1–1.8)	**0.019**	1.3 (1.0-1.7)	0.074
Adjuvant chemotherapy	0.8 (0.6-1.0)	0.059	0.5 (0.4–0.7)	**< 0.001**

BMI: body-mass index, ASA: American Society of Anesthesiologists, HR: hazard ratio, CI: confidence interval

## Discussion

This cross-sectional study found that patients with different ABO blood groups had similar survival after upfront resection of head PDAC. Furthermore, blood group was not found as a survival predictor. Only lymph node involvement, tumor differentiation (G3), and adjuvant chemotherapy were predictive of OS after surgery.

The results of the present study corroborate the findings of a monocentric study published in 2020 that showed that ABO blood types did not influence OS and DFS after pancreatectomy for PDAC [9]. Of note, the AB group was excluded (too small number of patients), while patients with neoadjuvant chemotherapy (26%) were included, which differs from the present study. The previous results are also concordant with two other studies where survival after resection did not differ between the ABO subgroups [15, 16]. Two other studies found different results [17, 18]. Khalil et al. found a better OS after pancreatoduodenectomy for periampullary cancers in group O compared to group A [17]. However, not only PDAC were included but also ampullary carcinomas, cholangiocarcinomas, and duodenal adenocarcinomas. Furthermore, in the subgroup analysis of PDAC patients, no difference in OS was found between group O and group A (HR 1.06, 95% CI 0.77–1.46, *p* = 0.724). Rahbari et al. showed a better long-term prognosis after resection for PDAC patients with group O compared to non-O group patients, but included pancreatoduodenectomies, distal pancreatectomies, and total pancreatectomies without subgroup analysis based on the operation type [18]. Moreover, only 53% of patients had R0 resections, which seems rather low compared to the R0 rates currently published in the literature.

Several hypotheses have been postulated on the potential mechanism played by ABO groups regarding cancer development. As ABO genes encode glycosyltransferases, which are involved in signaling and intercellular adhesion, it has been suggested that these functions promoting local tumor implantation might be altered based on the specific blood group [19]. Additionally, it has been shown that Lewis blood phenotype-negative patients (Lewis antigens are closely related to ABO blood groups) are not able to synthesize carbohydrate antigen 19−9 (CA 19−9) [20]. If CA 19−9 is considered as a tumor promoter, lack of circulating CA 19−9 in Lewis-negative patients might decrease angiogenesis, protein glycosylation, or selectin binding, therefore reducing cancer progression [21]. Another hypothesis is that the ABO gene is directly related to the level of circulating inflammatory mediators (e.g., TNF-α, E-selectin, or P-selectin) that play a role in the local inflammatory tumor environment [22–24]. The results of the present study and other previous studies on postoperative outcomes after PDAC resection suggest that ABO groups might have an influence on the development of pancreatic cancer but not on the survival after surgical resection.

Some limitations of the study need to be acknowledged. The retrospective design of the study includes a risk of collection errors and of bias due to missing data. Moreover, the relatively small number of patients within each blood group subtype might have had an influence on the non-significant results (type II error). Results of this study can only be applied to patients with PDAC who were operated upfront without preoperative chemotherapy. Results might be different in a population of patients who received neoadjuvant treatment, which warrants further studies including PDAC patients treated with neoadjuvant chemotherapy followed by surgery. Response and susceptibility to chemotherapy based on blood groups remain currently unknown. In addition, immunotherapy may also be integrated in the management of PDAC patients in the future, and the impact of ABO groups in this setting may be of particular interest. It should also be noted that the incidence of lymph node involvement (pN+) was not statistically significant between ABO groups in this cohort, but the p-value approached 5% (*p* = 0.058). This should be kept in mind when interpreting the data as lymph node involvement is an important independent predictor of OS.

In conclusion, this study including two Western centers showed that blood groups did not impact survival of patients with PDAC who underwent upfront pancreatoduodenectomy. In this international cohort, predictors of survival were associated with characteristics of tumor biology (lymph node involvement and tumor differentiation) and adjuvant treatment.

## Data Availability

No datasets were generated or analysed during the current study.
